# Beyond Color: Hybrid Vibrational–Electronic Broadband Coherent Anti-Stokes Raman Scattering for Molecularly Informed Digital Pathology

**DOI:** 10.1021/acs.analchem.6c01408

**Published:** 2026-06-20

**Authors:** Paul Ebersbach, Jayakrupakar Nallala, Neil Shepherd, Nick Stone, Julian Moger

**Affiliations:** † Department of Physics and Astronomy, 3286 University of Exeter, Exeter EX4 4QL, U.K.; ‡ Gloucestershire Cellular Pathology Laboratory, 156720 Cheltenham General Hospital, Cheltenham GL53 7AN, U.K.

## Abstract

We demonstrate that broadband coherent anti-Stokes Raman scattering (BCARS) on hematoxylin and eosin (H&E)-stained tissue generates a hybrid vibrational–electronic spectroscopic contrast arising from coupled Raman-active vibrations and chromatin–hematoxylin electronic resonances. This hybrid response, inaccessible to conventional spontaneous Raman or infrared microscopy due to fluorescence and substrate interference, transforms routine histology slides into sources of quantitative molecular information. The resulting spectra encode both Raman-active vibrations and resonance-modulated four-wave-mixing contributions arising from hemalum–chromatin interactions, thereby linking histological color contrast to quantitative, machine-readable spectral features. In breast tissue microarrays, we show (i) pixel-wise wavenumber-shift mapping that resolves subnuclear domains and highlights necrosis-associated nuclear shrinkage; (ii) phase-retrieved Raman-like spectra that separate hemalum-associated resonant features from nonpigmented contributions attributable to processing reagents; and (iii) nucleus-level discrimination of ductal carcinoma in situ (DCIS), invasive ductal carcinoma (IDC), and invasive lobular carcinoma (ILC) using a PCA–LDA workflow. This study is a feasibility demonstration rather than a clinical validation, but establishes electronically enhanced BCARS on routine H&E slides as a route to unlock archival repositories for molecular phenotyping and AI-enabled histopathology.

## Graphical abstract

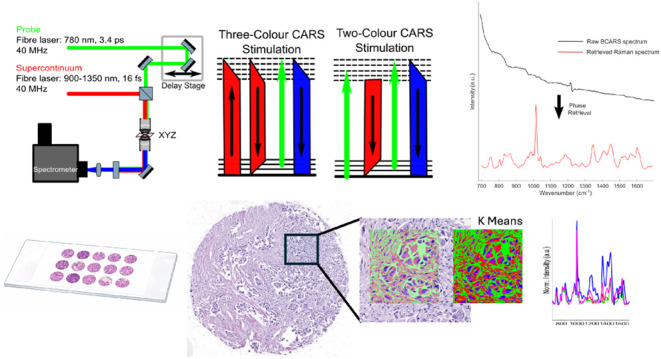

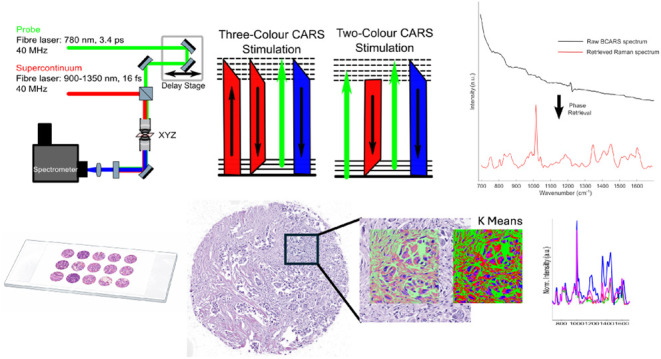

## Introduction

Histopathological microscopy remains a cornerstone of diagnostic and prognostic oncology, yet it is fundamentally limited to morphological and architectural analysis. Routine hematoxylin and eosin (H&E) staining, developed in the late 19th century, reveals cellular and tissue structure with remarkable clarity, allowing pathologists to identify and grade malignancies and identify diagnostic hallmarks of disease. Hematoxylin selectively binds to nuclear chromatin, staining it blue/purple, while eosin counterstains cytoplasmic and extracellular proteins pink. Despite its simplicity, this method continues to underpin virtually every histopathologic diagnosis worldwide.

In recent years, the advent of digital pathology and machine-learning has sparked scientific interest into the deeper biochemical and optical information encoded in H&E slides.^1^ Subtle variations in the hue, intensity, and texture of hematoxylin staining, long attributed to material density and staining variability, are now recognized as potential carriers of prognostic information.^2,3^ Quantitative image analysis studies have shown that the colorimetric and textural characteristics of hematoxylin-stained nuclei correlate with chromatin organization, DNA content, and nuclear texture, which in turn reflect molecular alterations driving tumor aggressiveness.^4–7^

Hematoxylin binding to nuclear material depends on the presence of negatively charged phosphate groups in DNA and RNA and on the electrostatic environment of chromatin-associated proteins. This affinity varies with chromatin condensation and nucleoprotein composition. In cancer cells, aberrations in chromatin organization, such as increased euchromatin, altered heterochromatin clustering, and nuclear envelope instability, modify dye accessibility and result in subtle yet reproducible differences in staining intensity and hue. These optical variations effectively act as proxies for molecular phenotype.^8^

Quantitative histomorphometry has shown that darker, more heterogeneous nuclear staining often correlates with aggressive behavior and poor clinical outcome, reflecting nuclear atypia, increased DNA ploidy, and transcriptional deregulation.^9^ Statistical descriptors of nuclear color distribution, such as the intensity range across the nuclear boundary, mean hematoxylin density, and pixel-level entropy have been identified as independent prognostic factors in breast and colorectal cancer.^10,11^ For example, nuclei exhibiting wider intensity gradients at their periphery, thought to reflect irregular chromatin condensation at the nuclear envelope, are associated with increased metastatic potential. Moreover, spatial heterogeneity of staining among neighboring nuclei has been linked to intratumoral clonal diversity, an established predictor of recurrence and treatment resistance.^12,13^

Machine-learning models trained on digitized H&E slides further confirm the diagnostic and prognostic value of hematoxylin signal variation in invasive cancer. Even relatively simple algorithms can stratify patient survival and predict molecular subtype by leveraging intensity- and texture-based features extracted from the nuclear hematoxylin channel. These features are closely related to the morphological hallmarks used by pathologists to assess tumor grade (such as nuclear pleomorphism, chromatin irregularity, and orientation disorder) and have been shown to be independently predictive of outcome in breast cancer and other carcinomas.^3,14^ Consequently, the apparent color and texture variations observed in nuclei are not technical artifacts, but quantitative optical readouts of altered nuclear architecture and epigenetic deregulation that underlie malignant progression and clinical aggressiveness. The hematoxylin signal provides only an indirect reflection of molecular state. Its sensitivity arises from chemical binding to chromatin, yet it lacks specificity for the biochemical constituents that define disease mechanisms. The information encoded in stain color is limited by the broad absorption spectrum of hematoxylin and by the optical constraints of bright-field microscopy. Therefore, while the subtle color of nuclei may reflect meaningful biological differences, it cannot directly quantify the molecular species responsible for them. This limitation motivates the exploration of molecular spectroscopic techniques that can extend the diagnostic power of histopathology beyond morphology.

Vibrational spectroscopy, i.e., Raman and infrared (IR), offer chemically specific contrast by probing the fundamental molecular vibrations of bonds within biological macromolecules. Vibrational spectra provide molecular fingerprints that reveal the relative abundance and spatial distribution of lipids, proteins, nucleic acids, and metabolites. When applied to tissue, these spectra can distinguish benign from malignant lesions, predict therapeutic response, and identify early biochemical transitions preceding morphological change.^15–20^ The ability to obtain such molecular information without staining or labeling makes vibrational spectroscopy an attractive complement to conventional histology.

However, the integration of vibrational spectroscopy into routine clinical workflows has been constrained by fundamental physical barriers. Spontaneous Raman spectroscopy, while chemically specific, is rendered unusable on H&E-stained slides by the intense fluorescence emission from hematoxylin and eosin dyes, which overwhelms the weak Raman signal. Infrared absorption spectroscopy is similarly incompatible due to the strong mid-IR absorption of standard borosilicate glass substrates and spatial resolution limitations that prevent subcellular nuclear imaging. While quantum cascade laser (QCL)-based IR systems have dramatically increased acquisition speeds compared with earlier Fourier-transform approaches, these platforms remain constrained by trade-offs between spectral bandwidth, spatial resolution, and substrate compatibility, and cannot access standard glass-mounted archival slides. These fundamental barriers have prevented the vast repositories of archived H&E-stained slides—representing decades of annotated clinical outcomes—from being accessible to molecular spectroscopic analysis, despite decades of compelling research demonstrating the diagnostic potential of vibrational spectroscopy.^21^

To bridge the longstanding gap between molecular spectroscopy and routine pathology, we introduce a spectral histopathology approach based on a hybrid nonlinear optical (NLO) contrast mechanism that couples vibrational and electronic resonances. Rather than extending existing techniques into new wavelength regimes, this approach exploits the intrinsic coupling between Raman-active vibrations and hematoxylin electronic resonances to generate a fundamentally new spectroscopic response within the BCARS framework. Both vibrational and electronic pathways contribute simultaneously to image contrast, generating hybrid signals that encode molecular vibrations and dye–chromophore interactions directly on conventional histology slides.

BCARS microscopy is a nonlinear Raman imaging technique that exploits four-wave mixing (FWM) to amplify weak vibrational signals, enabling chemical-fingerprint microscopy with millisecond pixel acquisition rates.^22^ Within the focal volume, narrowband and broadband laser pulses interact via sum- and difference-frequency generation to produce a coherent optical spectrum recorded at each pixel. The spontaneous Raman response interferes with the strong FWM field, producing oscillatory modulations (“ripples”) that encode vibrational information, which is recovered computationally through phase-retrieval algorithms.^23^ Critically, the coherent anti-Stokes detection scheme suppresses fluorescence background, and the nonlinear signal generation is largely insensitive to substrate absorption, enabling BCARS operation on standard glass-mounted H&E slides where spontaneous Raman and infrared spectroscopy cannot function.

Beyond its vibrational sensitivity, BCARS also responds to electronic absorption processes through electronically enhanced four-wave mixing (EE-FWM). When excitation wavelengths (or their nonlinear combinations) coincide with electronic resonances, the resulting FWM spectra are modulated by the sample’s absorption characteristics. Traditionally considered background, these modulations instead provide valuable biochemical insight, particularly in pigmented or stained tissues.

We recently showed that BCARS can operate in highly pigmented and H&E-stained specimens, including plant tissues dominated by strong electronic resonances, establishing that electronically enhanced four-wave mixing can be exploited as an information-bearing signal.^24^ The present work demonstrates that in clinical H&E histology, this electronically enhanced response is not merely a compatibility feature but a fundamentally new contrast mechanism. Hematoxylin is not simply a pigment but a nuclear-targeted chromophore whose electronic response is governed by chromatin chemistry and organization. By operating in a regime where both vibrational and electronic pathways contribute to the detected signal, BCARS generates hybrid spectra that encode chromatin–hematoxylin coupling. This transforms electronically enhanced FWM from background into a nuclear-specific spectroscopic reporter. The nonlinear spectra of hemalum-bound chromatin act as indirect molecular markers of nuclear composition, with the interplay between vibrational and electronic resonances producing rich, discriminative spectral features that enable nucleus-level phenotype classification.

Our approach, shown in Figure 1, leverages electronically enhanced BCARS to probe subtle variations in hematoxylin absorption that reflect its binding interactions with chromatin. Hematoxylin associates electrostatically with DNA and RNA phosphate groups, with affinity modulated by chromatin density/compaction and protein composition. In cancer cells, alterations in chromatin architecture (expanded euchromatin, disrupted heterochromatin, and nuclear envelope instability) affect dye accessibility and local optical response, generating reproducible spectral shifts that serve as optical proxies for molecular phenotype.

**Figure 1:**
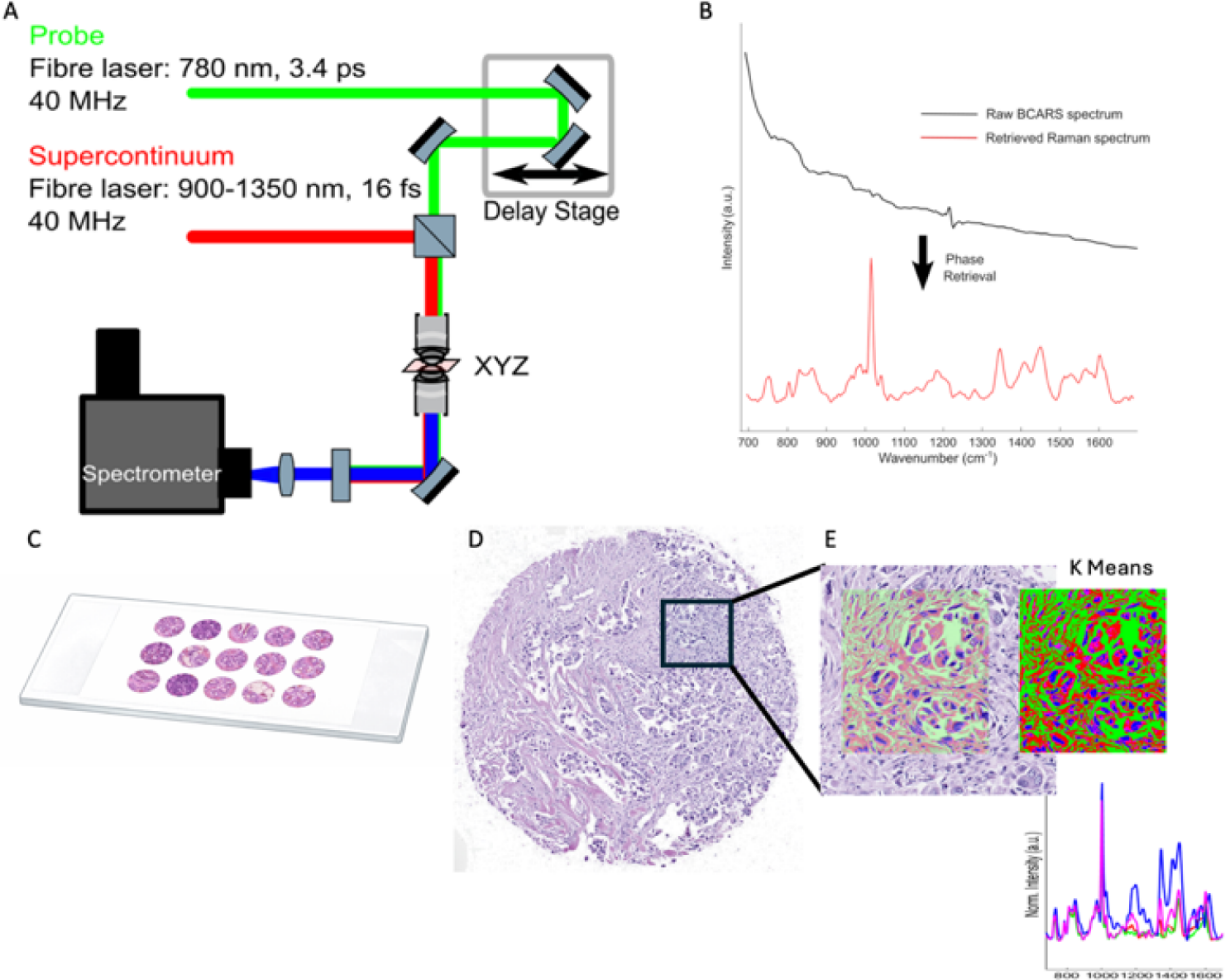
Schematic diagrams showing (A) the optical setup, (B) example result of phase-retrieval, (C) TMA sample configuration, (D) example H&E core image, and (E) example of k-means spectral clustering from nonlinear optical spectra from a region of interest within the core.

The combined Resonant-Raman and electronically enhanced FWM effects yield exceptionally strong optical signals, enabling pixel acquisition rates of ∼10 ms. While current acquisition times (∼26 min per 200 × 200 μm field of view) require further engineering optimization, whole-slide imaging is not a prerequisite for clinical implementation. Since the method operates on standard H&E slides, existing digital pathology algorithms can be used to automatically identify small regions of interest, within which the BCARS spectroscopic analysis is then performed. This targeted acquisition strategy integrates directly with conventional digital pathology workflows: the H&E image provides morphological guidance, and BCARS interrogates only the selected regions. Even if whole-slide BCARS acquisition were technically feasible at clinically acceptable speeds, the resulting hyperspectral data volumes would be prohibitively large and would themselves become the limiting factor. The ability to leverage established digital pathology algorithms for region-of-interest selection is therefore a practical strength of the approach rather than a limitation, and avoids the need for whole-slide molecular spectroscopy entirely.

By performing nonlinear spectroscopy directly on standard H&E slides, this approach integrates seamlessly into existing pathology pipelines and unlocks large archival slide libraries for retrospective molecular analysis. Access to such data sets will accelerate biomarker discovery and facilitate earlier, more precise disease detection.

Herein we demonstrate that BCARS microscopy, enhanced by electronic–vibrational coupling, offers a potentially powerful new contrast mechanism that unifies molecular spectroscopy with clinical histopathology. This hybrid modality has the potential to enhance the analysis of routine H&E-stained slides with multidimensional molecular data sets, augmenting AI-driven digital pathology with true biochemical content and paving the way for a new paradigm of spectral–morphological diagnosis that bridges biochemistry and morphology in cancer pathology.

In this work, we present a first feasibility study, establishing the concept of electronically enhanced BCARS as a hybrid spectral histopathology modality for conventional H&E slides. The primary aim is not clinical validation, but to demonstrate that nonlinear electronic–vibrational coupling generates reproducible, information-rich spectra from hematoxylin-stained nuclei, and that these signals encode biologically meaningful variation linked to nuclear composition and chromatin state. Using a pilot cohort of tissue microarray cores (N = 4 patients per cancer class), we show that this hybrid contrast mechanism enables robust spectral acquisition on standard glass slides, resolves intratissue biochemical heterogeneity, and supports proof-of-principle machine-learning discrimination of nuclear and tissue phenotypes at the single-nucleus level. The limited patient cohort reflects the pilot-scale nature of this study and is consistent with established practice for initial method validation in vibrational spectroscopy, where the objective is to demonstrate that a novel contrast mechanism produces discriminative spectral features prior to investment in large-scale clinical trials. Leave-one-patient-out cross-validation was employed to maximize robust performance estimation within this constraint. While the present study is not intended to define diagnostic sensitivity or clinical utility, it establishes the physical and biological basis of a previously unexplored contrast mechanism and demonstrates feasibility of cancer subtype discrimination from spectral signatures that are inaccessible to conventional spontaneous Raman or infrared microscopy. These results lay the groundwork for larger, statistically powered validation studies (target N ≥ 30 patients per class with independent test cohorts) aimed at determining how electronically enhanced BCARS can augment digital pathology with quantitative molecular information, enabling a new class of spectral–morphological biomarkers for cancer detection, risk stratification, and mechanistic insight.

An important interpretive point concerns the level at which classification is performed. Discrimination is carried out at the nucleus level, not at the patient or image level. Within any histologically defined region of interest, a proportion of nuclei will not carry the biochemical signature of the predominant cancer subtype, due to the presence of stromal, inflammatory, and morphologically normal cells intermingled with malignant nuclei. Nucleus-level misclassification therefore does not necessarily indicate classifier failure: it may reflect genuine biochemical heterogeneity of the tissue microenvironment. This is particularly relevant to the lower recall observed for DCIS (see Results), since DCIS spans a broad spectrum of nuclear grade and architectural pattern, and frequently contains morphologically normal epithelial cells within the same field of view. Intratumoral heterogeneity, driven by genetic and epigenetic diversity among coexisting cell populations, is well-established as a determinant of clinical outcome in breast cancer.^34^ In this context, the spatial distribution of spectrally distinct nuclei within a DCIS lesion may be reframed as a quantitative readout of intratumoral heterogeneity, which may carry independent prognostic significance. Formal sample size guidance for spectroscopic multivariate classifiers, such as that provided by Beleites et al.,^35^ recommends a minimum of 20–30 independent biological replicates per class for stable PCA–LDA models. Reaching this scale is the primary objective of future work. The current data, however, demonstrate clearly discriminative spectral features and consistent cross-validated performance, providing the positive feasibility evidence required to justify investment in larger cohort studies.

## Experimental Section

### Tissue Samples

Formalin-fixed, paraffin-embedded (FFPE) breast tissue cores (1 mm diameter) were obtained from a commercial tissue microarray slide with associated clinical annotations (US Biolabs, Maryland, USA). Cores representing invasive ductal carcinoma (IDC, n = 4 patients), invasive lobular carcinoma (ILC, n = 4 patients), ductal carcinoma in situ (DCIS, n = 4 patients), and normal breast tissue (n = 2 samples) were selected for BCARS hyperspectral imaging based on tissue quality, core integrity, and representative histological features as assessed from corresponding H&E-stained images. H&E staining was performed by the supplier using an automated stainer following a standardized protocol with the following reagents: Avantik 95% and 100% alcohols, Epredia Signature Series Clarifier 1 (ref. #7401) and Bluing Reagent R (ref. #7301), Optik Type 2 Hematoxylin (Gill II), and Optik Type 2 Eosin. Samples were mounted on Fisherbrand Superfrost Plus slides.

Regions of interest (ROIs) for BCARS imaging were identified from the corresponding H&E-stained images. Initial ROI selection was performed by the research team to maximize representation of the diagnostic pathology for each core by prioritizing areas with high tumor-cell density and characteristic histological features, while excluding artifacts, extensive necrotic areas, and nonrepresentative peripheral tissue. All selected ROIs were subsequently reviewed and verified by a pathologist to ensure accurate histological classification and diagnostic relevance. The 12 cancer cores used in the study, are shown in full, along with BCARS cluster maps of the ROIs used in the study are provided in the supplementary file (Figure S1).

### BCARS Microscope

A custom-built BCARS imaging system based on the configuration by Camp et al. and described in detail by Ebersbach et al, Anal. Chem 2025. Briefly a pump beam (770 nm; ∼16 mW, 3.4 ps pulses on-sample) and broadband Stokes beam (∼900–1350 nm; ∼9.5 mW, ∼16 fs pulses on-sample) are generated from two coseeded fiber lasers (Toptica Photonics, FemtoFiber pro), temporally superimposed via a superimposed via a motorized delay line (ThorLabs, MCM 3000) and coupled into an inverted microscope (Olympus, IX71) using a water-immersion objective lens (Olympus, UPlanSApo IR, NA = 1.2, ×60; controlled via ThorLabs, MCM 3000). &quot; Should read: &quot;Briefly a pump beam (770 nm; ∼16 mW, 3.4 ps pulses on-sample) and broadband Stokes beam (∼900–1350 nm; ∼9.5 mW, ∼16 fs pulses on-sample) are generated from two coseeded fiber lasers (Toptica Photonics, FemtoFiber pro), temporally superimposed via a motorized delay line (ThorLabs, MCM 3000) and coupled into an inverted microscope (Olympus, IX71) using a water-immersion objective lens (Olympus, UPlanSApo IR, NA = 1.2, ×60; controlled via ThorLabs, MCM 3000). Stage scanning is enabled via a xyz-piezo stage (Physik Instrumente, P-545 and E-727 controller) in submicrometer precision. The emitted light is collected and collimated with a ×60 objective lens (Olympus, LUCPlanFL N, NA = 0.7) and short-pass filtered (Semrock, Brightline 770SP; Chroma, HHQ765SP). The remaining anti-Stokes light is spectrally separated and focused on the central row of a CCD chip (IsoPlane 160, Princeton Instruments) with 10 ms/spectrum acquisition time.

BCARS hyperspectral imaging of H&E slides was performed at areas of around 195 × 200 μm with 500 nm resolution resulting in x-y-λ stacks of size 390 × 400 × 1340. This generated around 500,000 BCARS tissue spectra from all the normal cores (n = 1), all DCIS cores (n = 4) and 4 of each of IDC and ILC cores. To reduce day-to-day variation induced artifacts we tried to image a core of each group at 1 day and randomize the order of imaging.

### Data Preprocessing

Raman-like hyperspectral images were generated using a phase-retrieval workflow developed by Camp et al.^23^ and implemented as described in Ebersbach et al.^24^ Prior to phase retrieval, noise was reduced using singular value decomposition (SVD) to ensure stable reconstruction. K-means clustering was applied to segment each hyperspectral image into spectrally distinct regions, and clusters enriched in nuclei—the primary focus of this study—were selected for subsequent unsupervised multivariate analysis.

Data processing was performed in a custom MATLAB R2020a graphical interface. Cosmic rays were identified via spectral-spatial local-maximum detection and removed using inward spatial interpolation. Variance stabilization was achieved using an Anscombe transform to convert mixed Poisson and additive Gaussian noise into Gaussian-distributed noise. The transformed data were decomposed by SVD, and noise components were identified from their high-to-low frequency ratios in the 2D Fourier domain and removed following manual validation. An exact inverse Anscombe transform was applied to restore original scaling.

Phase retrieval was performed using a Hilbert transform with cover-glass spectra as the nonresonant background (NRB) reference. Residual multiplicative amplitude and phase-offset artifacts arising from pixel-wise NRB mismatch were corrected following the phase- and scale-error compensation procedure of Camp et al.

The imaginary spectral component was isolated, concatenated across the fingerprint and CH-stretch regions, and vector-normalized prior to spectral unmixing to ensure that analyses reflected differences in spectral shape rather than absolute intensity.

### Spectral Shift Processing

In pigment-rich samples, we often observe spectral-shifts affecting the entire spectrum. These shifts are corrected via a cross-correlation based algorithm and are also used as important optical measures that reflect the local frequency dependent absorption properties. To separate the shift information from other spectral shape information we applied a shift correction algorithm based on the finddelay.m function in MATLAB, which estimates the delay between two signals. As a reference signal we use the mean spectrum of the whole data set. The algorithm only works properly for spectra, which are similar to the reference spectrum (in this case the ER-enhanced hemalum spectral features), as the algorithm is based on cross-correlation. Pixel spectra which do not show a high similarity, normally give very extreme delay values, thus they can be eliminated via outlier search using isoutlier.m.

### Spectral Measure Extraction

Nuclei spectra were extracted via three cluster k-means applied on 2D median filtered data set for each hyperspectral image separately. To get insights into the spectral variability between different images in the masked nuclei regions we performed again k-means with 3 clusters, however this time using a fused data set as input. We obtain two clusters with quite strong ER-pigment features (red and green) and one cluster reflecting out-of-focus nuclei features with less strong ER boosted features (blue). We removed out-of-focus pixels from further analysis. Moreover, we only retained images that retained at least 50 remaining pixels following this approach.

### Unsupervised Multivariate Analysis

K-means clustering was used to get a general idea of the spectral variance and its spatial distribution. To get further insights, we use Principal Component Analysis (PCA) to decompose the data set with respect to its spectral variance. The first principal component possesses the highest variance in the data set and thus reflects the main spectral information. The second PC is orthogonal to the first one inside the spectral space and contains the second largest variance and so on. The spectral information is visualized in loading spectra, with positive loadings reflecting a positive correlation and negative loadings a negative correlation. The spatial information is shown via score maps.

### Nuclei Classification

A schematic of the process is shown in Figure 5a. Nuclei phenotype classification was performed by spatially segmenting individual nuclei to extract mean spectral profiles, which were subsequently used to discriminate between DCIS, IDC, and ILC using a principal component analysis–linear discriminant analysis (PCA–LDA) approach using the first 20 principal component score values. Nuclei were delineated using a Chan–Vese active contour segmentation algorithm applied to average intensity projections of the hyperspectral images.^25^ The segmented nuclei contained a median of 34.5 pixels per nucleus for DCIS (n = 404 nuclei; mean 50.3), 47.0 pixels for IDC (n = 626; mean 65.7), and 36.0 pixels for ILC (n = 457; mean 52.2), with all objects subject to a minimum size threshold of 20 pixels to exclude subnuclear fragments.

The images were then normalized and thresholded, to obtain initial segmentation masks, with morphological filtering to suppress noise. Contours were evolved in region-based mode, producing smooth nuclear boundaries without assuming specific edge gradients or shape priors. Resulting masks were refined, by filling internal holes and excluding objects outside predefined size limits, to remove small debris and large aggregates. Eight-connected components were used to define individual nuclei, and the final binary masks were used for all downstream analyses. Mean spectra were then calculated over the pixels contained within each nuclei and spectra aligned to a common Raman-shift axis to remove shift artifacts. Spectra were then processed using Savitzky–Golay smoothing (SVG) and standard normal variate (SNV) normalization applied on a per-nucleus basis to reduce baseline and intensity variability.

Classification was performed using a supervised PCA–LDA pipeline. The training data set included only cancer classes and was balanced by random under-sampling to match the minority class size. Principal component analysis (PCA) was used for dimensionality reduction, with the first 20 components. The LDA model was optimized by 5-fold cross-validation to maximize classification accuracy. Classification performance was assessed using confusion matrices and one-vs-rest receiver operating characteristic (ROC) curves with area under the curve (AUC) metrics calculated using the (held-out) test nuclei.

To assess robustness and guard against overfitting at small sample size, we additionally performed an exhaustive search across all 4^3^ = 64 possible combinations of held-out test images (one image per class), evaluating every possible patient-level partition. Mean AUC across the top four combinations was 0.961 (SD 0.007), and AUC exceeded 0.90 in 11 of 64 combinations. Cross-validated training accuracy was 95–97% across all top combinations. The low variance in AUC across independent partitions provides empirical evidence against overfitting. The summed confusion matrix across the top four combinations yielded per-class correct classification rates of 68.7% (DCIS), 85.5% (IDC), and 91.4% (ILC), with per-class mean AUC values of 0.976 (SD 0.033), 0.959 (SD 0.033), and 0.947 (SD 0.021) respectively.

## Results

### Broadband FWM Signal Generation in H&E Sections

To demonstrate the hybrid vibrational–electronic nature of the BCARS contrast mechanism, a preprocessed, non-phase-retrieved BCARS hyperspectral image of an H&E-stained invasive lobular carcinoma, showing necrotic tissue on the right-hand side, was segmented into six clusters by k-means clustering. The mean spectra of these clusters (Figure 2a) reveal three nuclei-associated clusters, two from non-nuclei tissue, and one from extracellular or background regions.

**Figure 2:**
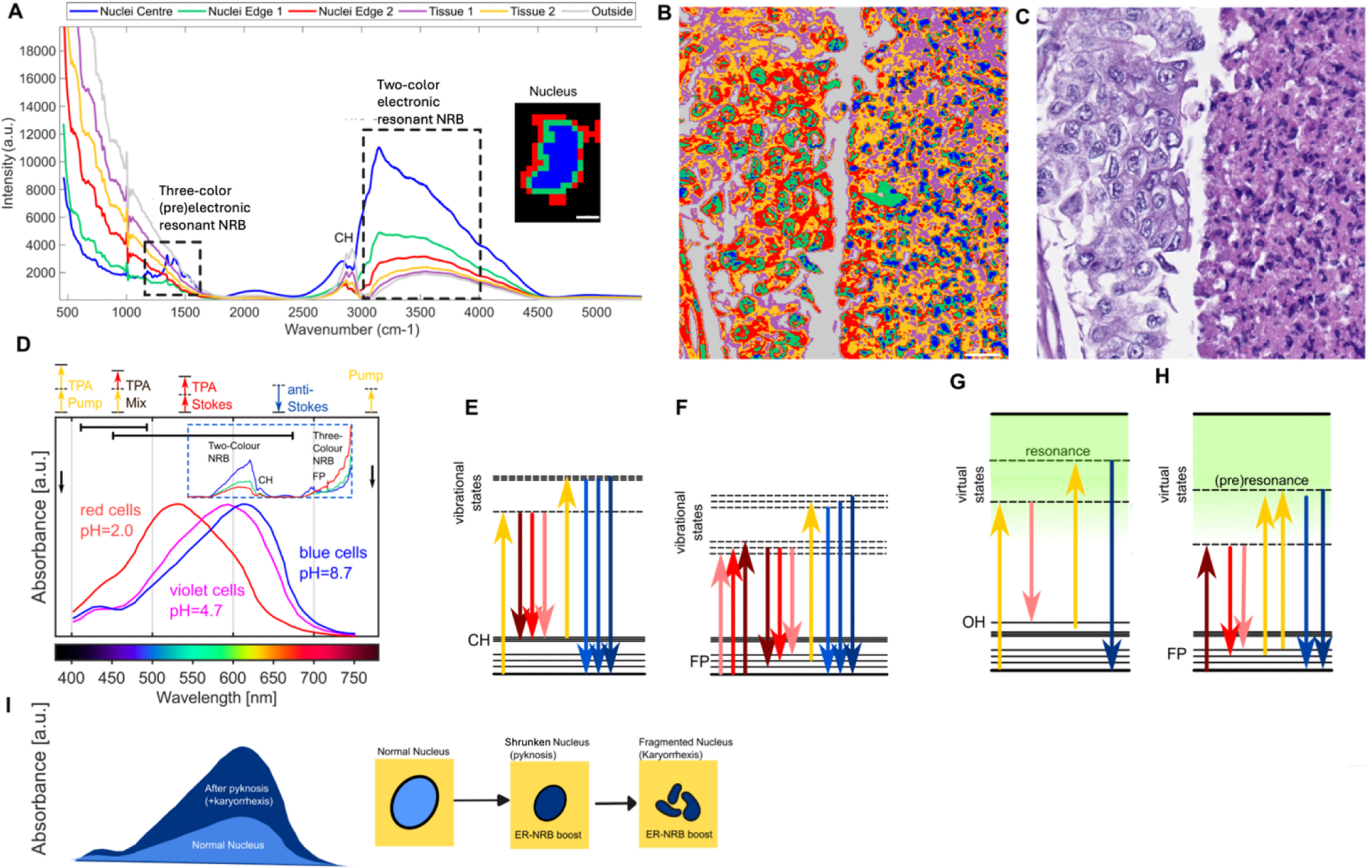
Hybrid vibrational–electronic BCARS signal generation in H&E-stained tissue. (A) K-means clustering of raw BCARS spectra from an ILC core: nuclear (blue, green, red), tissue (magenta, yellow), and background (gray) clusters. (B) Cluster map. (C) H&E image. (D) Hemalum absorbance at varying pH (from ref 32); inset: anti-Stokes range. (E–F) Conventional two- and three-color BCARS in nonpigmented regions. (G–H) Electronically (pre)­resonant two- and three-color excitation in hemalum-stained nuclei. (I) Enhanced NRB in pyknotic nuclei.

Across all clusters, both the fingerprint (500–1700 cm^–1^) and CH-stretching (2800–3000 cm^–1^) regions contain features arising from weakly or unstained “outside” areas (gray in Figure 2a). These spectra show distinct aromatic signatures consistent with residual xylene, an organic solvent used during H&E preparation, and a prominent band near 1000 cm^–1^ consistent with aromatic ring breathing (e.g., xylene residues) and/or sulfate-associated vibrations from alum-containing reagents.^26–28^ All slides were subjected to the supplier’s standard deparaffinization protocol prior to H&E staining, which includes sequential xylene immersion and graded alcohol dehydration steps. Any residual xylene present following this process would be expected to contribute uniformly across all tissue classes on a given slide; its spectral signature (most prominently the aromatic ring breathing mode near 1000 cm^–1^) is therefore unlikely to introduce class-specific bias into the PCA–LDA model. Consistent with this, the 1000 cm^–1^ band appears across all cluster types in Figure 3 rather than being enriched in any single cancer class.

**Figure 3:**
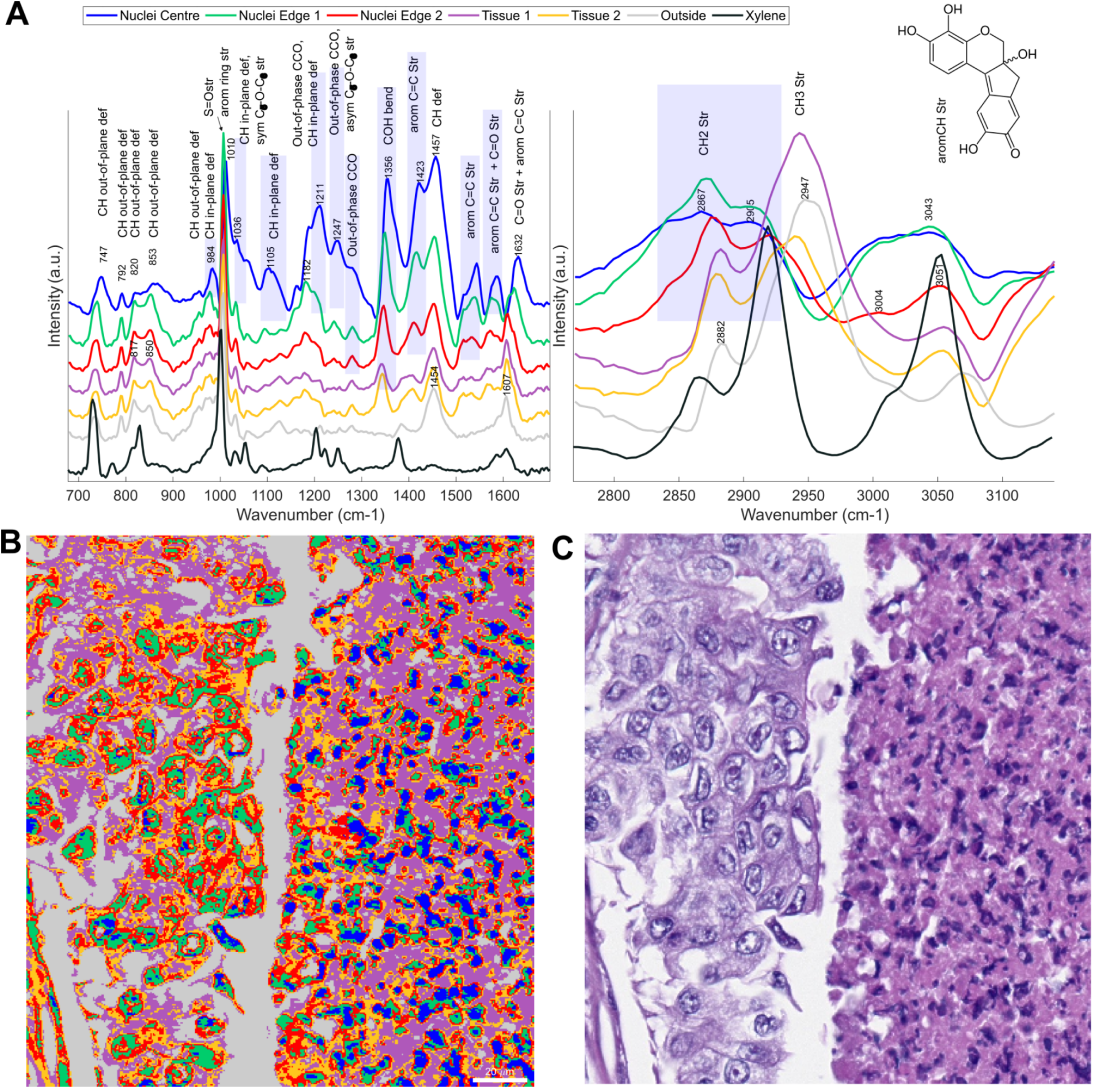
K-means clustering of a Raman-like BCARS hyperspectral data set of a H&E slide. (A) K-means mean cluster spectra separating HE image into 3 nuclei regions (blue, green, red) and two tissue (magenta, yellow) and one outside region (gray). A xylene spectrum is shown as reference (black). Nuclei-specific pigment features are highlighted in blue boxes. Peak assignments according to ref 35 are listed in full in the Supplementary Table S1. (B) Corresponding cluster map. (C) H&E transmission image.

The distorted profiles in the fingerprint region result from modulation by the nonresonant background (NRB), which amplifies Raman features through a three-color, four-wave-mixing (FWM) process (Figure 2f). In contrast, the CH-stretching response arises mainly from a two-color interaction (Figure 2e). The spectra from nuclear regions exhibit additional, strong features superimposed on this background—most prominently in the upper fingerprint region and the two-color NRB zone beyond the CH-stretching range. Their intensity increases from nuclei periphery to the center, producing a strong signal enhancement consistent with electronic (pre)­resonance amplification of the NRB and Raman components, generated by the hematein–aluminum complex bound to DNA.^29^ It is likely that Al^3+^ ions bridge the anionic hematein to negatively charged phosphate groups along the DNA backbone, forming the characteristic insoluble blue polymeric complex.^27^ Hematein is the oxidized form of hematoxylin produced via sodium iodate oxidation in Gill’s protocol,^26^ forms the basis of this complex, conventionally termed hemalum, which is adopted throughout this study.

Figure 2d shows the absorbance spectra of hemalum-stained HeLa cell nuclei.^30^ Depending on the pH, the absorbance maximum shifts from approximately 530 nm (strongly acidic conditions, red cells) to 600 nm (violet cells) and further to 610 nm (basic conditions, blue cells). The absorbance in the violet and blue regions therefore overlaps with the anti-Stokes signals generated in the two-color NRB region, resulting in strong electronic-resonance enhancements. This explains the highly modulated and amplified signal features observed in the central nuclear regions (Figure 2g).

Based on the electronic resonance window proposed by Wei et al.,^29^ the pump wavelength used here (770 nm) and, to a lesser extent, the Stokes wavelengths (900–1350 nm) fall within or close to electronic absorption bands associated with nuclear pigments. In particular, for violet-stained regions with an absorption maximum near 580 nm, the corresponding electronic resonance window has been suggested to extend to approximately 1080 nm. Under this framework, an electronic-resonance-mediated enhancement of the nonresonant background (NRB) in the two-color BCARS configuration is therefore plausible.

However, it is important to note that the definition and practical applicability of such electronic resonance windows remain a matter of active debate in the literature.^31^ Consequently, while the observed enhancement of anti-Stokes emission in the two-color NRB region is consistent with an electronic resonance or preresonance contribution, this interpretation cannot be considered definitive. In the higher-wavenumber fingerprint region, the enhanced anti-Stokes signals may arise from several nonexclusive mechanisms, including direct electronic (pre-)­resonance with the excitation fields, resonance mediated by the generated anti-Stokes photons themselves, or a combination of both processes (Figure 2H). Further spectroscopic or wavelength-dependent measurements would be required to unambiguously disentangle these contributions.

Notably, the strongest ER-NRB-enhanced features (blue clusters) are predominantly found in the nuclei of necrotic tissue (right-hand side of the image), corresponding to the darker regions visible in the HE image. These dark nuclei reflect nuclear shrinkage, or pyknosis, the irreversible condensation of chromatin during necrosis, followed by karyorrhexis, the fragmentation of the nucleus. This is likely represented by the irregular shapes of the dark nuclear regions. The increased accumulation of chromatin, and consequently of DNA and the hemalum complex, leads to the pronounced ER-NRB enhancement.

Moreover, BCARS provides a unique contrast arising from nonpigmented features that are not visible to the naked eye. This additional contrast may offer deeper insights into nuclear composition and structure. Toward the center of nuclear regions, we observe strong electronically enhanced FWM features, likely originating from hemalum bound to DNA.

### Chemical Information from BCARS Spectra

Figure 3 shows the k-means clustering results of the same data set after phase retrieval, producing Raman-like spectral features. Using the same six-cluster k-means decomposition as for the raw BCARS data, yields similar clustering results: three clusters correspond to nuclei, two to tissue regions, and one to the external area. This indicates that phase retrieval is not necessarily required as a preprocessing step when the goal is to identify the main spectral variances captured by simple k-means clustering. These dominant variances are largely influenced by the ER-NRB-enhanced features and therefore reflect the general absorption characteristics of the sample. However, to resolve subtle spectral differences in Raman bands or to enable more accurate peak assignments comparable to spontaneous Raman spectra, phase retrieval is required.

The Raman-like spectral bands can be broadly categorized into two groups: those originating from the nonpigmented regions and those from the electronic-resonance-enhanced hemalum-rich nuclear regions. Moving from the outer areas toward the nuclear centers, the proportion of hemalum-related spectral features gradually increases. The nonpigmented spectra exhibit partial overlap with xylene, for example at the aromatic ring band near 1010 cm^–1^, which could alternatively be attributed to S=O stretching vibrations from sulfates. Additional bands, such as the CH deformation band at 1457 cm^–1^, suggest the presence of other CH-rich (nonaromatic) compounds.

The hemalum-associated spectral features show good correlation with spectra from previous resonance-Raman studies (excitation at 488 nm) of Al­(III)–hematein complexes, whose peak assignments were used for comparison.^32^ In this study, a reference spectrum of hematein was also presented, showing only minor differences, most notably, a weak band at 646 cm^–1^ (Al–O stretch) unique to the Al complex. Another example using 532 nm excitation reported similar band characteristics,^33^ with strong features predominantly above 1300 cm^–1^.

Although the high-wavenumber CH-stretching region (2800–3100 cm^–1^) displays spectral variation within the tissue, it is likely to be heavily influenced by the paraffin waxing and dewaxing process. We therefore focus our tissue classification analysis on the fingerprint region. A further consideration is that the CH-stretching region in the raw (prephase-retrieved) spectra is dominated by the two-color electronically enhanced NRB signal visible in Figure 2A and F. The strong electronic resonance enhancement in this region means that the two-color signal is driven primarily by the absorption properties of the hemalum chromophore rather than by molecular CH-bond content, which complicates direct chemical interpretation. By restricting classification analysis to the fingerprint region of the phase-retrieved spectra, where vibrational assignments are well-established and resonance-Raman contributions are better characterized, we minimize the risk of incorporating processing artifacts or chromatically dominated contrast into the model.

### Spectral Shift as a Measure of Absorption

The application of principal component analysis (PCA) and linear discriminant analysis (LDA) to the nuclear spectra reveals that the most significant discriminating factors among nuclei are spectral shifts rather than intensity changes. We therefore apply a shift detection algorithm (see Experimental Section for details) to map spectral shifts at the pixel scale. Figure 4B presents four representative examples alongside the corresponding H&E-stained images, providing insight into the structural and chemical origins of the observed spectral phenomena.

**Figure 4:**
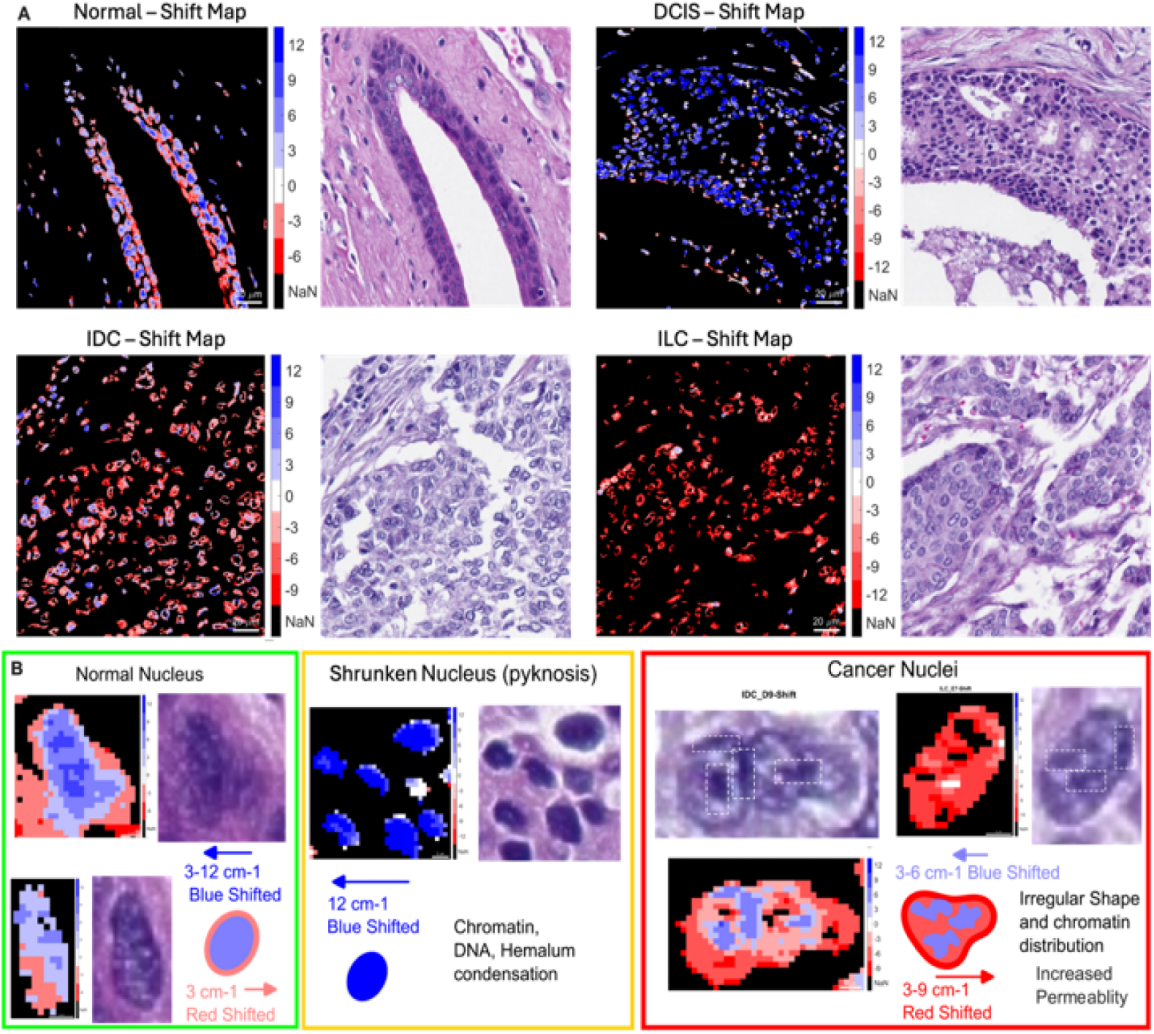
Wavenumber shift maps versus H&E transmission images of a normal, DCIS, IDC and ILC tissue. (A) Overview images. (B) Zoomed in single nuclei images and cartoons visualizing the wavenumber shift distribution in normal, shrunken and cancer nuclei.

Spectral shifts ranging from ±12 cm^–1^ with respect to the mean value are observed within nuclei regions across the four tissue classes. The shift-maps (Figure 4A) show close correlation with the contrast and texture patterns visible in the H&E images. Regions exhibiting blue-shifted spectra align with the dark blue-stained areas, whereas red-shifted regions coincide with the lighter blue to reddish areas. These correlations indicate that the observed spectral shifts reflect variations in optical absorption arising from heterogeneities in nuclear staining. In contrast to conventional H&E microscopy, BCARS imaging provides a more detailed and quantitative depiction of the optical absorption distribution within individual nuclei (Figure 4B).

In normal nuclei, blue-shifted regions are evenly distributed throughout the central nuclear area. In contrast, cancerous nuclei display smaller and spatially separated blue-shifted domains surrounded by more red-shifted regions toward the nuclear center. These red-shifted zones correspond to areas with lower hemalum concentration, which can be attributed to an irregular distribution of chromatin and DNA, or to increased nuclear permeability that leads to dilution of hemalum compared to normal nuclei.

In necrotic tissue, shrunken nuclei exhibit a more pronounced blue-shift relative to normal nuclei. This is consistent with an increased local concentration of chromatin, DNA, and consequently hemalum. Since necrotic regions are commonly observed in ductal carcinoma in situ (DCIS), we observe a significantly higher mean pixel-wise blue-shift in these areas compared to normal nuclei. Interestingly, certain cancerous tissues also exhibit blue-shifted nuclei associated with necrotic regions, in contrast to the red-shifted nuclei observed in non-necrotic cancerous tissue (e.g., ILC, right side of Figure 4B). When averaged across the full data set, the net shift is red rather than blue, despite the local presence of blue-shifted necrotic nuclei. This reflects the fact that non-necrotic cancerous nuclei (which are red-shifted relative to normal) constitute the majority of the data set and dominate the mean. The data set-mean shift is therefore not sensitive to the proportion of necrotic tissue in any given image, establishing this spectral measure as robust against variation in necrotic content across samples.

An important remaining question concerns the underlying origin of the observed wavenumber shifts. These may either result from changes in absorption amplitude, reflecting variations in DNA and hemalum concentration (light blue vs dark blue regions), or from intrinsic shifts in the hemalum absorption band position. The latter would imply changes in the hematein ionization or aluminum-complexation state, as well as alterations in the local chemical environment such as pH or binding interactions with the phosphate backbone of DNA.^30^

### Spectral Variance between Cancer Classes

To emphasize spectral features rather than spectral shifts, the following analysis was performed on shift-corrected spectra. Figure 5 presents the principal component analysis (PCA) of the shift-corrected fused data set. The first 20 principal components (PCs) account for 98% of the total spectral variance, with PC1, PC2, and PC3 explaining 41%, 21%, and 12%, respectively (Figure 5A). Spatial distributions of these components (Figure 5B) reveal pronounced and reproducible single-nucleus patterns across normal tissue and all cancer subtypes, including clear differences between peripheral and central nuclear regions and distinct signatures associated with shrunken nuclei.

**Figure 5:**
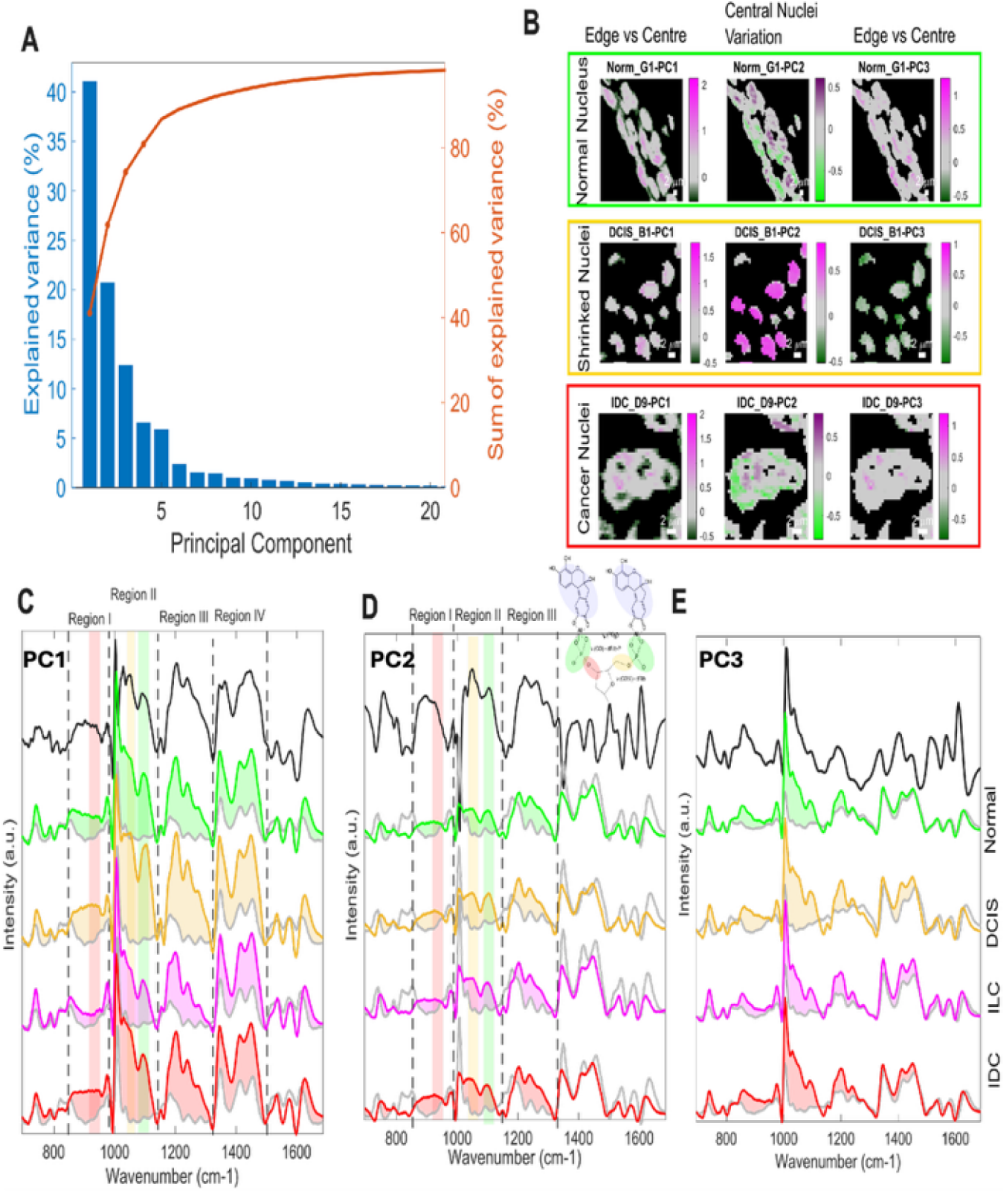
PCA of shift-corrected spectra. (A) Explained variance captured by the first 20 principal components. (B) Representative PC1–PC3 score-map zooms showing normal, shrunken, and cancer cell nuclei. (C–E) PC1–PC3 loading spectra (black) together with mean spectra extracted from pixels with extreme scores (>P99 and <P10) for normal (green), DCIS (yellow), ILC (pink), and IDC (red). For PC1 (C), mean scores at >P99 and <P10 were 2.8 and −0.3 (normal), 2.8 and −0.3 (DCIS), 2.4 and −0.4 (ILC), and 2.3 and −0.4 (IDC); for PC2 (D), 0.7 and −0.5 (normal), 0.8 and −0.3 (DCIS), 0.8 and −0.3 (ILC), and 0.8 and −0.3 (IDC); and for PC3 (E), 1.3 and −0.2 (normal), 1.2 and −0.5 (DCIS), 1.3 and −0.1 (ILC), and 1.2 and −0.2 (IDC).

To aid physical interpretation, Figure 5C–E show the loading spectra for PC1–PC3 (black traces) together with mean spectra calculated from pixels with extreme positive (>P99) and extreme negative (<P10) scores. The values given in parentheses (e.g., 2.8, −0.3) report the mean PCA scores of the >P99 and <P10 populations, respectively, and therefore quantify the degree of separation along each principal component, directly linking PCA axes to chemically meaningful spectral variations. To clarify this representation: each pixel in the hyperspectral image is assigned a score for each principal component, quantifying how strongly that pixel’s spectrum resembles the pattern described by the corresponding loading. Pixels with scores above the 99th percentile (>P99) are the locations where that spectral pattern is most strongly expressed; pixels below the 10th percentile (<P10) are locations where it is absent or reversed. The mean spectra extracted from these two extreme populations therefore represent the spectral character of the most and least “centre-like”, “shrinkage-like”, or “aromatic-enriched” pixels in each tissue class, as defined by each PC in turn. Comparing these extreme-score mean spectra with the loading directly confirms which spectral regions drive the variance captured by that component and validates that the loadings correspond to chemically real, spatially coherent differences within nuclei rather than noise or instrument artifact.

PC1 captures spectral variation between peripheral and central nuclear regions (Figure 5B). Its loading spectrum (Figure 5C) shows positive contributions across four regions: I (860–950 cm^–1^), II (990–1140 cm^–1^), III (1160–1320 cm^–1^), and IV (1320–1490 cm^–1^). The >P99 spectra exhibit systematically higher intensities across all regions compared to <P10 spectra for normal tissue as well as DCIS, ILC, and IDC. The comparable score separations across tissue classes (e.g., ∼2.8 versus −0.3) indicate that PC1 represents a global nuclear contrast axis rather than a disease-specific signature, consistent with increased chromatin density or enhanced electronic-resonance contributions in nuclear interiors.

PC2 reflects spectral variations within central nuclear regions, with the highest scores observed in shrunken nuclei, suggesting its potential as a marker of nuclear shrinkage. The PC2 loading spectrum (Figure 5D) shows enhanced contributions from regions I–III, while region IV and the ∼1000 cm^–1^ aromatic band show little or no contribution. The >P99 and <P10 spectra display more modest intensity differences and narrower score separations than PC1, indicating that PC2 captures secondary spectral shape variations associated with nuclear compaction rather than dominant intensity changes.

Regions I and II likely include contributions from the phosphate–deoxyribose backbone of DNA, where the hematein–aluminum complex may bind via phosphate coordination. These possible DNA-backbone features could be spectrally separated from hemalum-specific features via MCR in shrunken nuclei regions of DCIS tissue (see Supplementary Figure S2). In shrunken nuclei, increased local DNA and chromophore density may enhance surrounding vibrational modes through electronic-resonance four-wave mixing (ER-FWM), or via more direct electronic coupling of the phosphate backbone to the chromophore complex. Region III overlaps with characteristic DNA bands, including the cytosine and thymine ring vibration near 1243 cm^–1^.

PC3, like PC1, captures differences between nuclear edges and centers but with a distinct chemical emphasis. Its loading spectrum (Figure 5E) is dominated by the ∼1000 cm^–1^ band, indicating increased nonpigmented aromatic-associated vibrations in central nuclear regions. The >P99 and <P10 spectra show smaller yet reproducible differences and greater heterogeneity across tissue types, suggesting that PC3 reflects finer-scale biochemical or structural heterogeneity within nuclei.

Overall, the combined use of PCA score maps, loading spectra, and extreme-score mean spectra shows that the leading PCs correspond to physically interpretable modes of nuclear spectral variation: PC1 captures dominant, phenotype-independent nuclear contrast, PC2 highlights changes associated with nuclear shrinkage, and PC3 encodes subtler biochemical heterogeneity within nuclear interiors. It should be noted that the derivative-like line shape visible near 1000 cm^–1^ in the PCA loading spectra of Figure 5 is a residual artifact of the phase-retrieval algorithm, arising from imperfect NRB reference matching at this wavenumber. It does not reflect a genuine spectral feature of the tissue and does not affect the classification analysis, which is performed on the full phase-retrieved spectra after standard normalization.

### Nuclei-Level Classification

Classification was performed at the nuclear rather than pixel level to account for the heterogeneous cellular composition of each field of view, which includes stromal, immune, and nonmalignant epithelial cells in addition to cancerous nuclei. Phase-retrieved Raman-like spectra were used to suppress nonresonant background contributions while preserving chemically interpretable vibrational bands and resonance-enhanced intensity modulations arising from chromatin–hematoxylin interactions.

Spectra were segmented and averaged over individual nuclei using Chan–Vese active contour masks (Figure 6A), followed by outlier rejection to exclude partial nuclei, debris, and out-of-focus regions. This aggregation strategy reduces contributions from noninformative subnuclear structural variation while retaining nuclear-scale spectral signatures linked to chromatin organization and dye microenvironment. To prevent class imbalance bias, the training data set was balanced by random undersampling to match the minority class size. Nuclei were sampled from N = 4 patients per class (DCIS, IDC, ILC), with leave-one-patient-out cross-validation ensuring that performance estimates reflect interpatient variance rather than artificially inflated intrapatient correlations. This validation strategy guarantees statistical independence between training and test nuclei at each fold.

**Figure 6:**
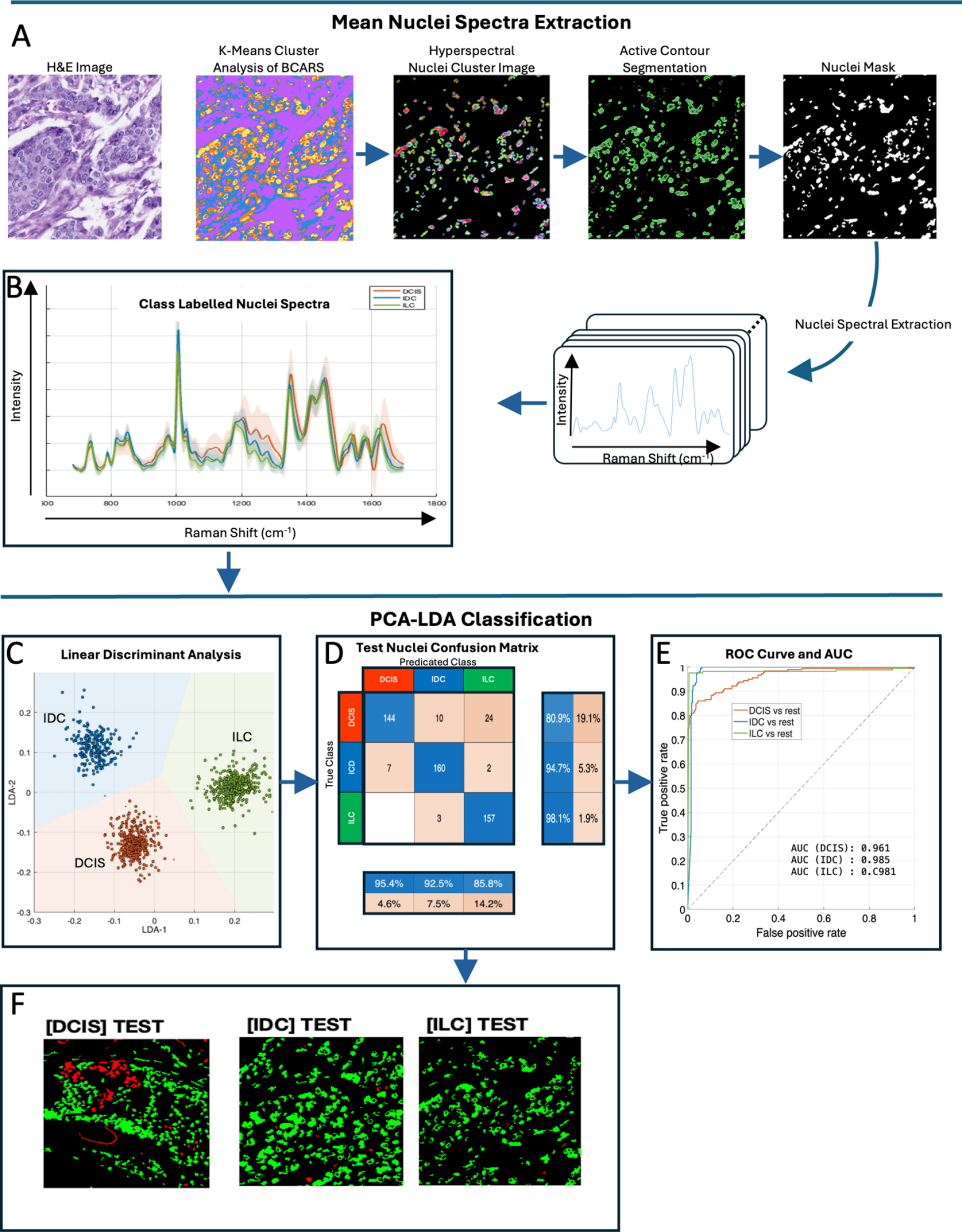
Nucleus-level PCA–LDA classification of breast cancer subtypes. (A) Segmentation and spectral averaging workflow. (B) Mean spectra of classified nuclei (DCIS, blue; IDC, red; ILC, pink; shading: ±1 SD). (C) LDA1–LDA2 projection of test nuclei (N = 507; leave-one-patient-out CV). (D) Confusion matrix: 90.9% accuracy. (E) ROC curves; AUC: 0.96–0.99. (F) Spatial map: green, correctly classified; red, unclassified/nonmalignant nuclei.

A PCA–LDA classifier was trained to discriminate DCIS, IDC, and ILC nuclei using the first 20 principal components as input features. The model achieved an overall classification accuracy of 90.9% on 507 test nuclei, with class-specific sensitivities of 80.9% (DCIS), 94.7% (IDC), and 98.1% (ILC). Corresponding precision values were 95.4%, 92.5%, and 85.8%, respectively. One-vs-rest receiver operating characteristic analysis yielded area under curve (AUC) values of 0.96 (DCIS vs rest), 0.99 (IDC vs rest), and 0.98 (ILC vs rest), demonstrating excellent class separability across all cancer subtypes.

Examination of LDA discriminant function coefficients reveals the spectral origins of classification performance. LDA1, which accounts for the majority of between-class variance and primarily separates DCIS from the invasive classes, loads most heavily on principal components that emphasize spectral variance in regions II–III (990–1320 cm^–1^), corresponding to hemalum-associated resonance-enhanced features and phosphate-backbone contributions. LDA2, which discriminates between IDC and ILC, shows dominant contributions from principal components weighted toward region I (860–950 cm^–1^) and the aromatic band near 1000 cm^–1^, reflecting differences in nonpigmented molecular environments and dye microenvironment accessibility. This feature attribution confirms that classification is driven by chemically interpretable variations in chromatin–hematoxylin interactions and nuclear composition rather than nonspecific intensity differences or instrumental artifacts.

Figure 6C shows the distribution of test nuclei projected into the discriminant subspace defined by the first two linear discriminants (LDA1 and LDA2). Each point represents an individual nucleus, positioned according to its predicted class relative to the decision boundaries of the trained model (indicated by shaded regions). Figure 6D presents the corresponding confusion matrix. The strong diagonal dominance confirms robust discriminative performance, with specificities exceeding 92% for all classes. To clarify the interpretation of Figure 6C: LDA1 (horizontal axis) primarily separates DCIS from the two invasive classes—the DCIS cluster (blue) occupies a distinct horizontal range from both IDC (red) and ILC (pink), which overlap substantially along this axis. LDA2 (vertical axis) then discriminates between IDC and ILC within the invasive class—IDC and ILC are vertically offset from one another, while DCIS occupies an intermediate vertical position. The separation is therefore sequential rather than simultaneous: LDA1 first isolates DCIS, and LDA2 then resolves IDC from ILC within the invasive group. This sequential structure is a property of how LDA constructs orthogonal discriminants to maximize between-class variance in order, and is reflected in the axis labels [LDA1, LDA2] added to the updated Figure 6C.

Off-diagonal misclassifications were nonuniformly distributed: DCIS showed the lowest sensitivity (80.9%) with misclassifications split between IDC (5.6%) and ILC (13.5%), whereas IDC and ILC exhibited higher classification fidelity with minimal cross-confusion (1.2% and 1.9%, respectively). The atypical DCIS-to-ILC misclassification pattern may reflect spectral overlap between high-grade DCIS with necrosis and certain ILC phenotypes, both of which can exhibit altered chromatin accessibility and nuclear irregularity. Alternatively, these cases may represent sampling at architectural transition zones or mixed histological regions inherent to tissue microarray cores. The high precision for DCIS (95.4%) despite lower sensitivity indicates that false-positive DCIS assignments are rare; the primary limitation is incomplete capture of the DCIS spectral phenotype space at this sample size.

The lower recall for DCIS (68.7% in the exhaustive partition analysis) is biologically interpretable rather than simply a classifier deficiency. DCIS is the most heterogeneous of the three classes, spanning a broad spectrum of nuclear grade and architectural pattern and frequently containing morphologically normal epithelial cells within the same imaging field. By contrast, ILC is characterized by a uniform population of small discohesive cells, which yielded the highest recall (91.4%) across partitions, consistent with greater biochemical homogeneity. Critically, classification is performed at the nucleus level: within any histologically defined region, a proportion of nuclei will belong to stromal, inflammatory, or morphologically normal cell populations, and their misclassification does not represent a failure of the spectral discriminant. The spatial distribution of spectrally distinct nuclei within a DCIS lesion may therefore be reframed as a quantitative readout of intratumoral heterogeneity, which may carry independent prognostic significance. This hypothesis connects to the broader framework linking nuclear spectral heterogeneity to epigenetic plasticity and clonal diversity.^34^

Mean spectra of correctly classified nuclei (Figure 6B) reveal class-specific differences concentrated in regions II–III (990–1320 cm^–1^), consistent with variations in hemalum-associated features and phosphate-backbone contributions observed in the PCA loading spectra (Figure 5). Standard deviations reflect intraclass spectral heterogeneity arising from chromatin compaction states and dye accessibility.

Spatial mapping of classified nuclei (Figure 6F) demonstrates that unclassified nuclei (shown in red) in test images correspond predominantly to noncancerous cell types inherent to the tumor microenvironment, as expected. The classifier correctly identifies spatially localized clusters of cancer-type-specific nuclei within heterogeneous fields of view, supporting the hypothesis that electronically enhanced BCARS encodes cell-type-discriminative information through chromatin–hematoxylin coupling. It is important to note that each field of view inherently includes a mixture of cell types, both malignant and nonmalignant. Thus, even partial classification of cancerous nuclei can meaningfully indicate the presence and proportion of cancer-specific cells within a region. In principle, this ratio could serve as a quantitative metric for tumor burden or histological grade, although establishing clinically relevant thresholds will require significantly larger patient cohorts and correlation with clinical outcomes.

Several limitations warrant acknowledgment. The current pilot-scale data set (N = 4 patients per class) limits assessment of interpatient biological variability and generalization to broader populations. Formal power analysis for multivariate spectroscopic classifiers does not follow standard parametric frameworks; learning-curve-based approaches are recommended instead,^34^ with a minimum of 20–30 independent biological replicates per class required for stable PCA-LDA models in spectroscopic applications; a threshold our current cohort does not yet meet.

The observed DCIS misclassification rate may reflect genuine biological heterogeneity, histological mixed-grade regions within cores, or sensitivity to staining protocol variance, which cannot be fully disentangled at this sample size. Additionally, all samples were prepared using a single standardized staining protocol (Gill’s hematoxylin); robustness to interlaboratory staining variation, slide age, and alternative hematoxylin formulations remains to be established. Performance on truly independent validation cohorts from different institutions and with varied clinical presentations is required to assess generalizability. These constraints motivate validation on larger, multisite cohorts with standardized clinical annotation, diverse histological presentations, and longitudinal outcome data to determine whether spectral nuclear phenotypes correlate with clinical end points such as recurrence risk or therapeutic response.

## Conclusions

By integrating vibrational and electronic resonances in a coherent nonlinear optical framework, we have demonstrated that broadband BCARS transforms standard H&E-stained tissue into a source of hybrid spectroscopic information inaccessible to conventional molecular imaging methods. The resulting spectra encode both Raman-active molecular vibrations and chromatin–hematoxylin electronic coupling, creating a fundamentally new contrast mechanism that bridges molecular composition and nuclear phenotype. This hybrid response provides chemically grounded, machine-readable features that extend digital pathology beyond morphology and stain color into quantitative molecular space.

BCARS images of nuclei exhibit intense contrast attributable to the hematein–aluminum (hemalum) complex, whereas surrounding tissue regions display spectral characteristics consistent with nonpigmented molecular components also present in unstained specimens. These include organic CH-rich compounds (CH deformation at 1450 cm^–1^), aromatic or unsaturated moieties (C=C stretching at 1600 cm^–1^ and a 1000 cm^–1^ aromatic band, likely from xylene), and contributions from aluminum sulfate (Al_2_(SO_4_)_3_) used as a mordant in Gill’s hematoxylin formulation (notably the S=O stretch near 1000 cm^–1^).

Strong four-wave-mixing backgrounds in the two-color region and pronounced Raman responses in the three-color region indicate that the nuclear BCARS signal is electronically resonance-enhanced, probing absorption-linked properties of the sample. These resonances are particularly prominent in shrunken nuclei characteristic of necrotic tissue, likely reflecting chromatin condensation and increased hemalum density.

We analyzed spectral variations across nuclei extracted from cancerous (invasive lobular carcinoma, ILC; invasive ductal carcinoma, IDC), (ductal carcinoma in situ, DCIS), and normal breast tissues using *k*-means clustering. Principal component analysis (PCA) of the fused hyperspectral Raman-like data sets revealed that spectral variance primarily arises from systematic frequency shifts in fingerprint bands, consistent with electronic-resonance-enhanced pigment effects previously observed in chloroplast regions of plant tissues. Cross-correlation-based spectral shift correction allowed quantification of pixel-wise deviations relative to the data set mean, distinguishing red- and blue-shifted regions. Comparison with corresponding H&E transmission images revealed a clear correspondence between red–blue spectral shifts and red–blue coloration in the histological images, though BCARS contrast provided substantially higher spatial and chemical resolution. At subcellular scale, BCARS maps resolved chromatin compartmentalization, identifying pigment-rich, blue-shifted domains within cancerous nuclei surrounded by red-shifted regions of lower density.

To extend beyond pixel-level analyses, we implemented a nucleus-level classification pipeline combining PCA and LDA. Averaging spectra over segmented nuclei reduced local structural noise and enhanced chemically representative signatures. This model achieved robust discrimination among normal, DCIS, IDC, and ILC nuclei, exhibiting strong diagonal dominance in the confusion matrix despite limited training data. Correctly classified nuclei displayed reproducible hybrid vibrational–electronic motifs reflecting chromatin organization and pigment binding chemistry.

These findings demonstrate that BCARS imaging of conventional H&E slides provides a quantitative, label-preserving platform for probing hybrid vibrational–electronic tissue signatures. The approach bridges molecular spectroscopy with histopathology, offering a machine-readable extension to traditional staining. While the current data set is limited, the results highlight the potential for objective diagnostic and prognostic assessment in clinical oncology, warranting validation in larger, statistically powered data sets.

A practical consideration for translation is that H&E is not a single, fixed stain. Laboratories employ different dye formulations, mordants, staining times, and mounting media, and slide appearance can further drift over years to decades due to oxidation, and dye fading. Such intersite and temporal variability is a well-known confounder for computational pathology models trained on bright-field color, as identical tissue phenotypes can map to different color distributions across protocols and epochs. Importantly, this variability strengthens rather than undermines the motivation of the present work. Electronically enhanced BCARS probes a hybrid vibrational–electronic response linked to chromophore chemistry and its microenvironment, yielding quantitative spectral features that are not solely defined by color (RGB) appearance. We therefore anticipate that, in larger cohorts, robustness across staining methods and slide ages could be achieved by combining these physics-linked features with domain adaptation strategies (such as transfer learning or feature-level alignment), in which representations learned on one slide set are adapted to new protocols using calibration data. While systematic multisite validation is beyond the scope of this feasibility study, establishing this hybrid contrast on routine H&E slides is a necessary first step toward cross-protocol, molecularly informative histopathology.

## Supplementary Material



## Notes

Raman band assignments (Table S1); bright-field transmission images of all 12 cancer tissue microarray cores used in this study, together with the corresponding BCARS k-means cluster maps of the regions of interest selected for hyperspectral imaging (Figure S1); multivariate curve resolution (MCR) decomposition of shrunken nuclei spectra in DCIS tissue (Figure S2) (PDF)
