# Supplementary material for: Beyond Color: Hybrid Vibrational–Electronic Broadband Coherent Anti-Stokes Raman Scattering for Molecularly Informed Digital Pathology
Source: Anal Chem. 2026 Jun 20;98(25):18759–73. doi: 10.1021/acs.analchem.6c01408 (PMC13325440; doi:10.1021/acs.analchem.6c01408)
Supplement: Supplementary file 1 [file ac6c01408_si_001.pdf]

Supporting Information

Beyond Colour: Hybrid Vibrational–Electronic Broadband Coherent Anti-Stokes Raman Scattering for Molecularly Informed Digital Pathology.

Paul Ebersbach[1], Jayakrupakar Nallala[1], Neil Shepherd[2], Nick Stone[1], Julian Moger*[1]

1. Department of Physics and Astronomy, University of Exeter, Exeter, EX4 4QL, UK

2. Gloucestershire Cellular Pathology Laboratory, Cheltenham General Hospital, Cheltenham, GL53 7AN, UK

*Corresponding Author email: J.Moger@exeter.ac.uk

ABSTRACT

This document provides additional data and analysis supporting the findings reported in the main manuscript. Table S1 presents Raman band assignments for the key spectral features identified in the BCARS spectra of H&E-stained tissue. Figure S1 shows bright-field transmission images of all 12 cancer tissue microarray cores used in this study, alongside the corresponding BCARS k-means cluster maps of the regions of interest selected for hyperspectral imaging, providing a complete visual record of the tissue material analyzed. Figure S2 presents multivariate curve resolution (MCR) decomposition of shrunken nuclei spectra acquired from DCIS tissue, offering further insight into the underlying spectral components associated with this morphological feature.

RAMAN BAND ASSIGNMENTS

The Raman-like BCARS spectra of H&E-stained tissue contain contributions from both non-pigmented tissue components and electronically resonance-enhanced hemalum-rich nuclear regions. To aid spectral interpretation of the k-means cluster spectra presented in Figure 3, Table S1 provides a comprehensive list of observed peak positions and their corresponding molecular vibrational assignments. Assignments are based on published resonance-Raman studies of Al(III)–hematein complexes[35] and, where relevant, include alternative attributions to non-pigmented species such as xylene residues and aluminium sulphate mordant. This reference table enables the reader to relate the spectral features highlighted in Figure 3A to specific molecular vibrations and to distinguish hemalum-associated bands from processing-reagent contributions.

	BCARS (cm-1)	Raman @ 488 nm<br><br>Hm:AI (III) 1:1 (cm-1)9	Assignment9
Hematein-Aluminium-Complex	(1632)	(1634)	(C=O str + arom C=C)
	1588	1581	arom C=C + C=O str
	1543	1549	arom C=C str
	1423	1434	arom C=C str
	1356	1384	COH bend
	1280	1258	Out-of-phase CCO str
	1247	1246	Out-of-phase CCO str, asym C5-O-C6 str
	(1211)	1216	Out-of-phase CCO stretch, CH in-plane deformation
	1105	1131	CH in-plane deformation
	1036	1053	CH in-plane deformation, symmetric C5 –O–C6 stretch
	1010	1006	Mixed in-phase ring stretch and in-plane ring bend, CH in-plane deformation
	984	973	CH out-of-plane deformation, CH in-plane deformation
	853	875	CH out-of-plane deformation

	829	830	CH out-of-plane deformation
	792	774	CH out-of-plane deformation
	747	747	CH out-of-plane deformation
Outside<br><br>(non-stained region, gray spectrum in figure 3b)			
	1607		C=C str
	1457		CH def
	1211		
	1010		Aromatic Ring Str/ S=O str from SO42-
	850		
	817		
	792		

Table S1. Peak positions (cm $^{-1}$) and molecular vibrational assignments for the spectral features identified in the BCARS k-means cluster spectra of H&E-stained breast tissue (Figure 3A). Assignments are based on resonance-Raman spectra of Al(III)–hematein complexes reported in reference 35.

DCIS, IDC AND ILC CORES

To ensure transparency and reproducibility, Figure S1 presents the full H&E-stained bright-field transmission images of all 12 cancer tissue microarray cores used in this study (4 DCIS, 4 IDC, and 4 ILC), together with the corresponding BCARS k-means cluster maps of the regions of interest (ROIs) selected for hyperspectral imaging. ROIs were chosen to maximise representation of diagnostic pathology within each core, prioritising areas of high tumour-cell density and characteristic histological features while excluding artefacts and non-representative peripheral tissue. All ROI selections were reviewed and verified by a pathologist. Presenting the full core images alongside the imaged sub-regions allows the reader to assess ROI placement relative to overall tissue architecture, evaluate the proportion of each core sampled by BCARS, and verify that selected regions are representative of the annotated diagnosis.

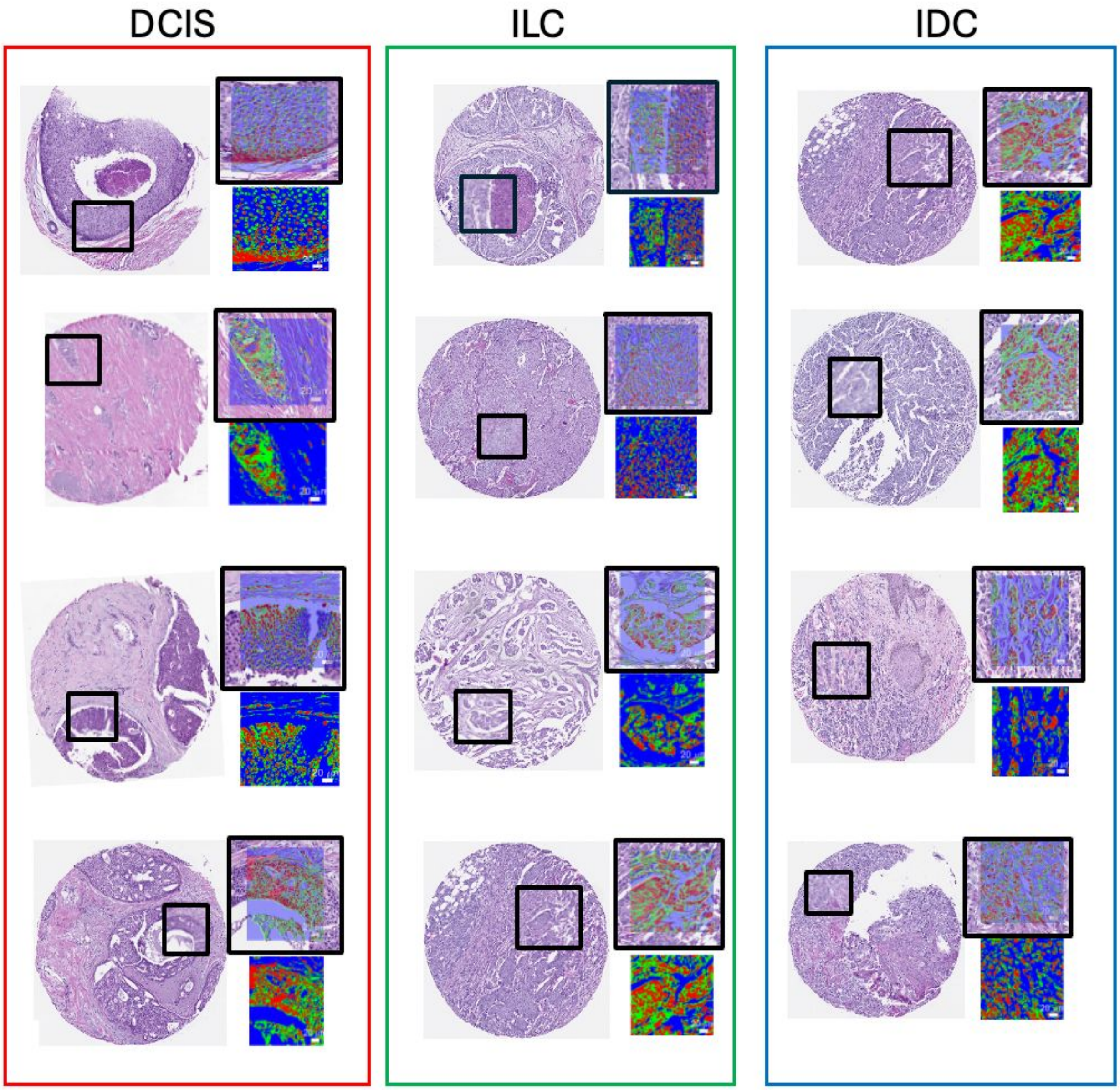

Figure S1: H&E-stained bright-field transmission images of all 12 breast cancer tissue microarray cores (DCIS, n = 4; IDC, n = 4; ILC, n = 4) with corresponding BCARS k-means cluster maps indicating the regions of interest selected for hyperspectral imaging.

MCR DECOMPOSITIONS POSSIBLY EXTRACTS DNA VIBRATIONAL FEATURES IN SHRUNKEN NUCLEI

We applied MCR decomposition on DCIS, the core with the highest Norm-DCIS score values to get further insight into these unique spectral pattern (figure S2). We obtain 2 MCR components separating hemalum spectral features from possible DNA-backbone features already speculated above. These are the PO2- stretch at 1103 cm^{-1} and CO stretching vibrations at 925 cm^{-1} and 1048 cm^{-1} This result is a further indeed that we not only access strong pigment features but might be even able to spectrally resolve the chemical surrounding.

The two MCR components might reflect different ratios of the chromophore vs. DNA-backbone feature signal boost, which might be dependent on the orientation and/or structure (e.g. structural changes due to DNA fragmentation[11]) of the hemalum-DNA complex and/or a contribution of non-DNA bounded hemalum. However, we have to emphasize that further studies are needed to really proof the appearance of DNA vibrational features inside the BCARS spectra e.g. possibly allowing to use the hemalum-complex as DNA stress sensor.

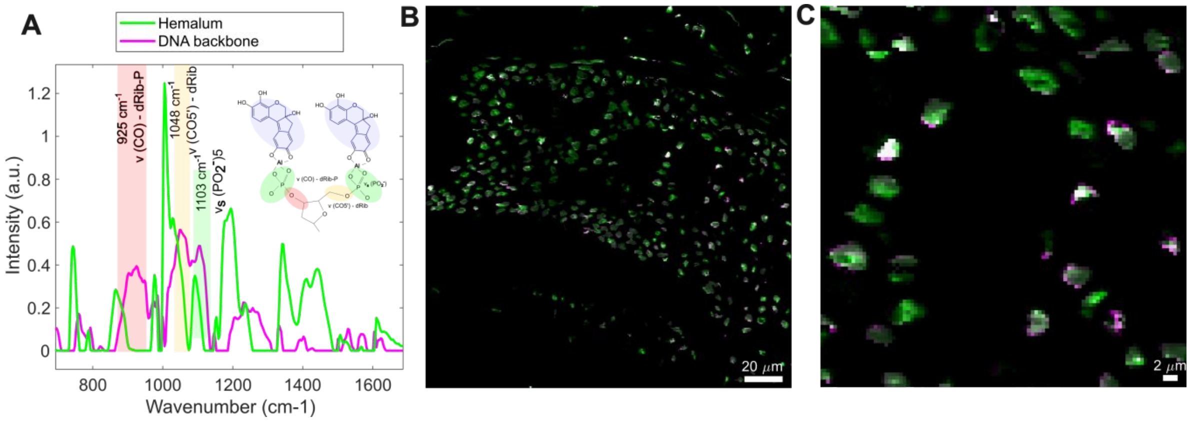

Figure S2: Multivariate curve resolution (MCR) decomposition of shrunken nuclei spectra in DCIS tissue. (A) MCR analysis separating phosphate–deoxyribose DNA backbone contributions from hemalum-specific spectral features in regions of nuclear shrinkage (B-C), demonstrating that DNA-backbone vibrational modes can be spectrally distinguished from chromophore-associated bands in regions of high chromatin and hemalum density.
